# Intra-Articular Injection of Bone Marrow Concentrate for Patellofemoral Osteoarthritis Treatment: Preliminary Results Using a New Tibial Endplate Sample Under Ultrasound Guidance

**DOI:** 10.3390/bioengineering12111150

**Published:** 2025-10-24

**Authors:** Alain Silvestre, Sébastien Caudron, Aymeric Rouchaud, Vladimir Borodetsky, Lionel Pesquer, Carlos Ferrer González-Adrio, Benjamin Dallaudière

**Affiliations:** 1 Musculoskeletal Radiology Department, Clinique du Sport, 33700 Mérignac, France; alain.silvestre@me.com (A.S.); lionelpesquer@gmail.com (L.P.); 2 Radiology Department, CHU Dupuytren, 87000 Limoges, France; sebcaudr@gmail.com (S.C.); aymeric.rouchaud@gmail.com (A.R.); zivova1982@gmail.com (V.B.); 3 Hospital HM Málaga, Av. de Carlos Haya, 121, Cruz de Humilladero, 29010 Málaga, Spain; cferrergonzal.ezadrio@gmail.com

**Keywords:** bone marrow concentrate, patellofemoral osteoarthritis, knee joint, ultrasound guidance, intra-articular injection

## Abstract

Introduction: Patellofemoral osteoarthritis (PFOA) remains a therapeutic challenge with few effective non-surgical options. Objective: The aim of this study was to evaluate the feasibility, safety, and preliminary outcomes of ultrasound (US)-guided tibial endplate aspiration and intra-articular injection of bone marrow concentrate (BMC) in patients with isolated PFOA. Methods: In this retrospective case series, seven consecutive patients with symptomatic PFOA unresponsive to conservative therapy were treated with US-guided tibial endplate aspiration followed by intra-articular BMC injection. Clinical outcomes were assessed with the Western Ontario and McMaster Universities Osteoarthritis Index (WOMAC) at baseline and 12 months. MRI with T2 mapping was performed to evaluate cartilage structure. BMC composition was analyzed, including colony-forming unit fibroblast (CFU-F) assays. Results: The procedures were feasible in all cases, and no adverse events occurred. WOMAC scores improved significantly from 21.7 ± 17.3 at baseline to 9.0 ± 9.3 at 12 months (*p* = 0.030). MRI showed a mean relative increase of 25.4% ± 43.5% in healthy cartilage volume, though this was not statistically significant (*p* = 0.49). Correlation analyses revealed no consistent association between clinical response and cellular composition, including estimated MSC dose. Conclusions: This small retrospective series suggests that US-guided tibial endplate aspiration and intra-articular BMC injection are safe, technically feasible, and may provide clinical benefit in isolated PFOA. Larger controlled studies are needed to confirm these preliminary findings.

## 1. Introduction

Patellofemoral osteoarthritis (PFOA) is a common cause of anterior knee pain, particularly in middle-aged and older adults [1]. Despite its prevalence, PFOA is often underdiagnosed and undertreated compared with tibiofemoral osteoarthritis (OA) [2]. Conventional conservative therapies—such as physical therapy, nonsteroidal anti-inflammatory drugs (NSAIDs), and intra-articular injections of corticosteroids or hyaluronic acid—typically provide only limited or short-term relief [3]. For patients who do not respond to these interventions and are not candidates for surgery, regenerative strategies are increasingly being explored.

Bone marrow concentrate (BMC) has emerged as a promising orthobiologic option for knee osteoarthritis. Its therapeutic potential is attributed to mesenchymal stem cells (MSCs), cytokines, and growth factors that contribute to tissue repair and modulation of inflammation [4]. Intra-articular injection of BMC has shown encouraging results for symptom relief and functional improvement in knee osteoarthritis, with several studies reporting clinically meaningful outcomes [5,6]. Traditionally, bone marrow is aspirated from the iliac crest. However, the tibial plateau has been proposed as a less invasive alternative, with evidence suggesting that although tibial aspirates may contain fewer nucleated cells, their biological quality and regenerative potential are comparable to iliac-derived samples [7,8,9].

Ultrasound (US) guidance improves both the precision and safety of bone marrow aspiration and intra-articular injections [10]. Intra-articular bone marrow concentrate (BMC) injections have been widely studied for tibiofemoral osteoarthritis, but their application in isolated patellofemoral osteoarthritis (PFOA) remains uncommon. To date, only one clinical study has specifically evaluated BMC for PFOA, using aspirates harvested from the posterior iliac spine under US guidance [6]. Other reports have assessed bone marrow mononuclear cells (BMMCs) in combination with arthroscopy [11], yet no investigation has described BMC injections derived from the tibial endplate in PFOA patients. Although the tibial plateau has been validated as a reliable alternative to the iliac crest for bone marrow aspiration in general knee OA [9], this approach has never been reported for PFOA. Therefore, to the best of our knowledge, this is the first study to describe the feasibility and clinical outcomes of US-guided BMC injections using aspirates from the tibial endplate in isolated PFOA

The aim of this study was to present preliminary clinical results of a novel US-guided technique for intra-articular BMC injection in patients with isolated PFOA, using aspirates obtained from the tibial endplate.

## 2. Materials and Methods

### 2.1. Patients and Clinical Assessment

This single-center retrospective study was conducted between June 2017 and June 2018 and included seven consecutive patients referred to our institution from sports medicine and orthopedic departments. All patients had persistent symptomatic anterior knee pain due to PFOA and were treated with intra-articular (IA) BMC injection after failure of conservative management (excluding surgery and stem cell therapy). The cohort included four men and three women, with a mean age of 50.1 years (median, 51.5; range, 37–68 years). The study was approved by the institutional ethics board (reference number 06-2019.2.) and registered with the Agence Nationale de Sécurité du Médicament. Written informed consent was obtained from all participants.

Inclusion criteria were:

- Persistent, non-traumatic PFOA for more than three months.
- Normal radiographs with MRI evidence of cartilage alterations.
- Age between 30 and 70 years.

Exclusion criteria were:

- Pregnancy, active smoking, diabetes, or ongoing infection.
- Previous IA treatments (corticosteroids, hyaluronic acid, PRP, MSCs, or BMC).
- Previous surgical treatment of the knee.
- Concomitant tibiofemoral osteoarthritis (assessed by MRI).
- Additional IA or surgical treatments during follow-up.

Analgesic use was recorded. Level 1 analgesics (e.g., paracetamol) were not evaluated, while oral steroidal and non-steroidal anti-inflammatory drugs, as well as level 2 and 3 analgesics, were assessed. Patients were instructed to contact the radiology department in case of any symptoms. Each follow-up visit included clinical evaluation and adverse event assessment. Clinical status was assessed at baseline (M0) using the Western Ontario and McMaster Universities Osteoarthritis Index (WOMAC) for lower limbs [12]. The study was designed as a preliminary feasibility investigation, focusing on technical viability and safety rather than statistical power. These results were intended to guide the design of future randomized controlled trials.

### 2.2. MRI Evaluation

All patients underwent MRI at baseline (M0). Scans were performed in the supine position with full limb extension using a 1.5 Tesla MRI scanner, between 3:00 p.m. and 5:00 p.m., under identical room conditions (temperature: 18 °C) to minimize diurnal and thermal variations in cartilage [13].

Conventional sagittal, coronal, and axial sequences were acquired in strictly orthogonal planes to the knee joint. T2 mapping sequences were acquired in the axial plane, aligned with the maximal patellar diameter to follow an anatomical landmark of the patellofemoral joint. The MRI protocol, including T2 mapping sequences, is detailed in Table 1. Mapping was performed in the same plane for each patient. T2 sequences were assessed on a GE^®^ workstation (General Electric Medical Systems, Waukesha, WI, USA) using semiautomated software. All images were anonymized. Based on literature and consultation with GE^®^ engineers, the threshold for normal T2 values was set at 40 ms [14,15,16]. This conservative cutoff ensured exclusion of fibrocartilage regardless of cartilage depth. T2 mapping was considered positive for healthy cartilage when values ranged from 0 to 39 ms. Images were analyzed in consensus by two radiologists, and only volumetric data from healthy cartilage were reported.

### 2.3. BMC Sampling, Preparation, and Injection

**Figure 1:**
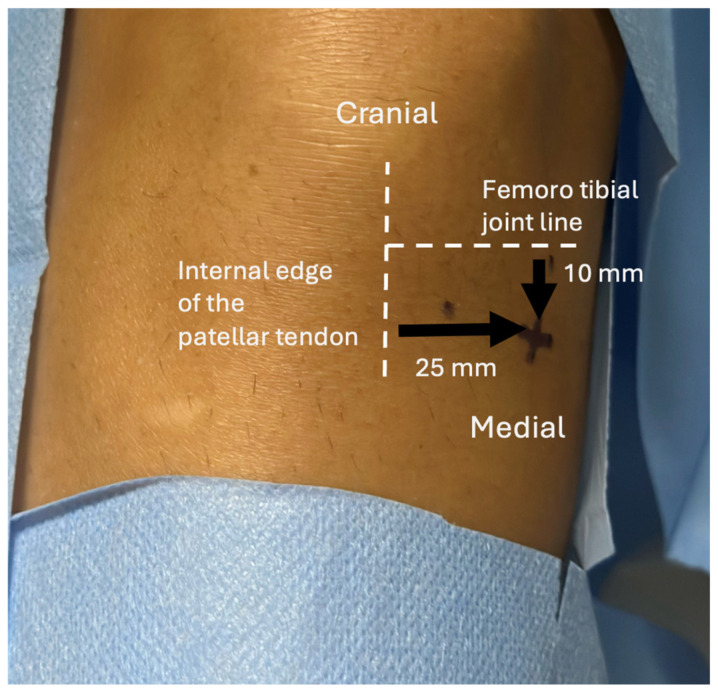
Recommended bone marrow aspiration site from the medial tibial plateau. The entry point is located 10 mm distal to the femorotibial joint line and 25 mm medial to the internal edge of the patellar tendon.

**Figure 2:**
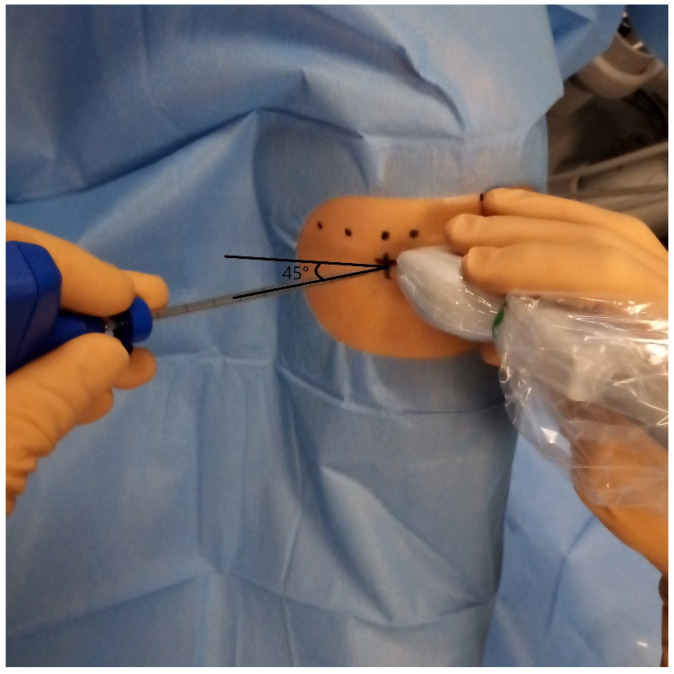
Ultrasound-guided puncture of the medial tibial endplate for bone marrow aspiration.

Procedures were performed by two musculoskeletal radiologists with 8 and 25 years of experience. Bone marrow aspiration (BMA) and IA injections were performed on the symptomatic knee during the same session. After local anesthesia with 10 mL of 1% lidocaine (Xylocaine^®^, Pierre Fabre, France; with intramuscular 21-gauge needle, Microlance™ 3, size: 0.8 × 40 mm, Becton Dickinson, Franklin Lakes, NJ, USA), BMA was obtained from the medial tibial epiphysis using a 13G harvesting cannula (BMHN1304VX, Argon Medical Devices, Frisco, TX, USA) under US guidance (17-MHz linear probe, EPIC 7, Philips Healthcare, Amsterdam, The Netherlands). The puncture site was located 25 mm medial to the patellar tendon and 10 mm below the femorotibial joint line, with a 45° angle toward the midline to avoid endplate injury (Figure 1 and Figure 2). These figures illustrate the anatomical landmarks and the feasibility of tibial endplate aspiration under ultrasound guidance. A total of 20 mL of bone marrow was aspirated into a syringe preloaded with 2 mL anticoagulant (80% ACD-A, 20% heparin 5000 UI/mL). The sample was centrifuged at 250× *g* for 12 min using a commercial kit (Hy-Tissue BMC, Fidia Farmaceutici S.p.A., L.P.Ponte Fabbrica, Italy). The buffy coat was carefully aspirated to avoid red blood cells, yielding 6–12 mL of BMC.

### 2.4. Cell Counting and Colony-Forming Unit Fibroblast (CFU-F) Assay

A 1 mL aliquot was reserved for cytology, including total cell count, platelet and leukocyte concentrations, and fibroblastic colony-forming units (CFU-F). The concentration of leukocytes (including lymphocyte subpopulations), platelets, and red blood cells in the bone marrow concentrate (BMC) was determined using a 3-differential hematology analyzer (Rayto RT-7600, Rayto Life and Analytical Sciences Co., Ltd., Shenzhen, China). For each patient, the total number of these cells administered was calculated by multiplying the concentration per milliliter by the injected BMC volume. To estimate the mesenchymal stromal cell (MSC) content, a colony-forming unit fibroblast (CFU-F) assay was performed according to Castro-Malaspina et al. (1980) [17], with minor modifications. Briefly, 1 × 10^6^ nucleated cells (corresponding to total leukocytes quantified by the analyzer) were seeded in triplicate into 6-well culture plates and maintained in DMEM-LG supplemented with 10% fetal bovine serum and 1× penicillin-streptomycin at 37 °C in 5% CO_2_. After 14 days, colonies (>50 cells) were fixed, stained, and counted. The CFU-F frequency was expressed as colonies per 10^6^ nucleated cells. The total MSCs injected were then estimated by multiplying the number of nucleated cells delivered by the CFU-F frequency, a commonly accepted CFU-F–based approach to approximate MSC dose. According to the literature, CFU-F are widely accepted as a functional surrogate for MSC quantity [15,18].

### 2.5. BMC IA Injection

**Figure 3:**
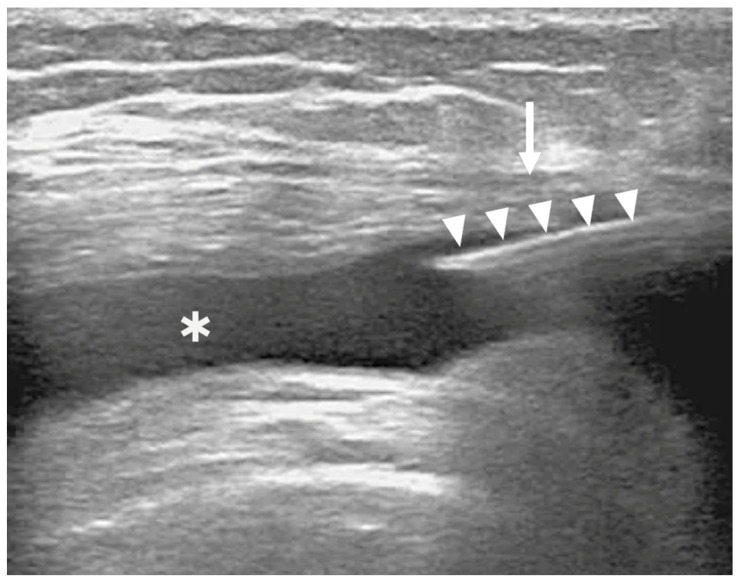
Ultrasound image showing intra-articular BMC injection in the knee. The arrowheads indicate the needle; the asterisk marks the suprapatellar pouch; the arrow points to the medial patellofemoral ligament.

IA injection was performed 30 min later under US guidance using a 21G needle inserted into the medial suprapatellar pouch (Figure 3). This figure demonstrates the technical feasibility and accuracy of intra-articular delivery of BMC. Proper IA positioning was confirmed in real time. Patients were monitored for 2 h post-procedure, allowed to walk unassisted, but advised to avoid strenuous activity for one month.

### 2.6. Twelve-Month Clinical and MR Follow-Up

Patients were reassessed 12 months after injection. WOMAC scores were collected, and MRI scans were repeated under the same technical conditions as baseline.

### 2.7. Statistical Analysis

All statistical analyses were performed using GraphPad Prism version 10.0.0 for Windows (GraphPad Software, Boston, MA, USA; Available online: www.graphpad.com). The assumption of normality for continuous variables was verified using the Shapiro–Wilk test. Categorical variables were expressed as absolute frequencies and percentages. Continuous variables were summarized as mean ± standard deviation (SD) and median and interquartile range (IQR). Paired comparisons between baseline and 12-month outcomes were performed using the paired *t*-test or the Wilcoxon signed-rank test, as appropriate. Correlation analyses were conducted using Pearson’s correlation coefficient, with two-tailed *p* values < 0.05 considered statistically significant.

## 3. Results

### 3.1. BMA and BMC Cell Counts

The cellular composition of BMC was analyzed for each patient. Samples contained platelets (653.1 ± 730 × 10^6^), leukocytes (63.7 ± 76.4 × 10^6^), mononuclear cells (MNCs, 2.16 ± 1.66 × 10^6^), fibroblastic colony-forming units (CFU-F, 254.9 ± 408.1 × 10^6^), and few erythrocytes (10.8 ± 17.4 × 10^6^). Detailed patient characteristics, BMA/BMC volumes, and cell counts are presented in Table 2.

### 3.2. Clinical Outcomes

**Figure 4:**
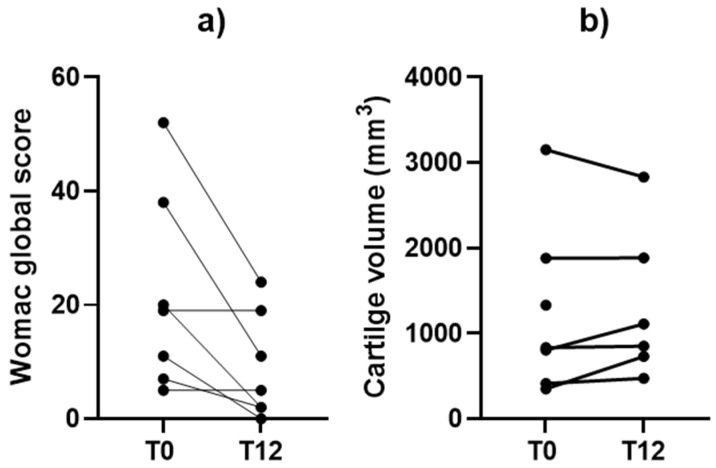
Individual changes in clinical and imaging outcomes at baseline (T0, months 0) and at 12 months (T12). (**a**) Global WOMAC scores: each line represents one patient’s trajectory between baseline and 12 months. (**b**) Cartilage volume measured on MRI: individual patient trajectories from baseline to 12 months are shown.

**Figure 5:**
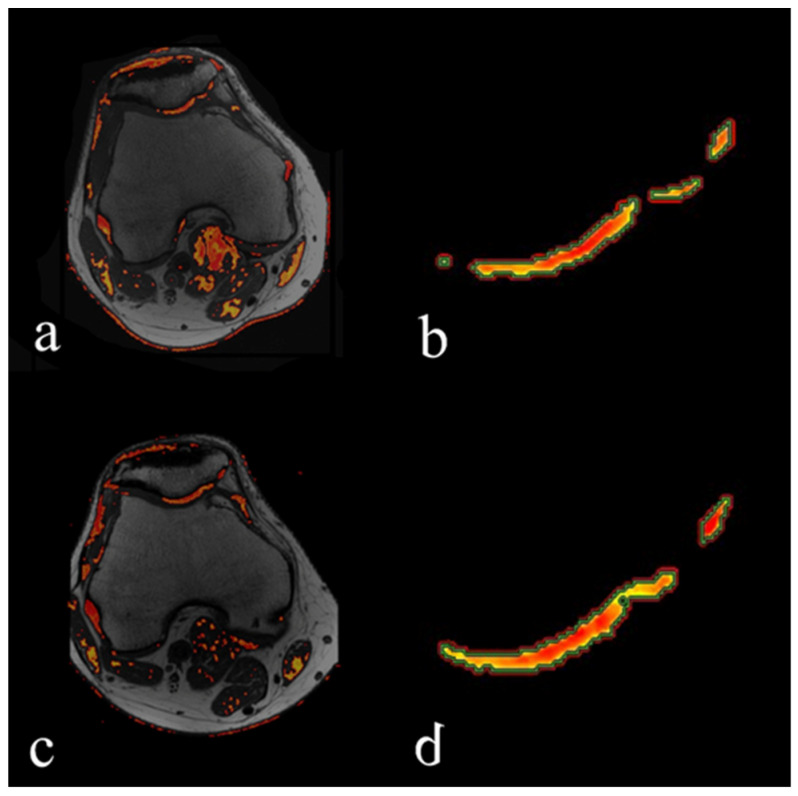
MR imaging follow-up in a 37-year-old patient at baseline (M0, (**a**,**b**)) and at 12 months (M12, (**c**,**d**)). The volume of healthy cartilage was estimated at 352 mm^3^ at M0 on T2 mapping (**a**,**b**) and at 731 mm^3^ at M12 (**c**,**d**). Images (**b**,**d**) are magnified views of the patellofemoral junction in (**a**,**c**), respectively.

Clinical and cartilage volumetric outcomes are shown in Figure 4, with representative MRI images shown in Figure 5. Mean WOMAC scores improved significantly from 21.7 ± 17.3 at baseline (T0) to 9.0 ± 9.3 at 12 months (T12) (paired *t*-test, *p* = 0.030). Cartilage volume increased by an average of 25.4% ± 43.5%, corresponding to a mean gain of 74.8 ± 248.6 mm^3^; this difference was not statistically significant (Wilcoxon signed-rank test, *p* = 0.49). Although cartilage gains were observed in five patients, no consistent pattern of structural improvement was confirmed at the group level. Notably, one patient demonstrated a 107.6% increase in healthy cartilage volume. Considering that annual cartilage loss in OA is typically estimated at ~4% [19,20], these findings suggest a potential trend toward structural stabilization.

Exploratory correlations were performed using Pearson’s coefficient to assess associations between patient-related factors, injected cell components, and clinical or imaging outcomes. Age showed no correlation with cartilage volume change (r = 0.02; 95% CI, −0.80 to 0.82; *p* = 0.97; *n* = 6) but correlated negatively with WOMAC improvement (r = −0.79; 95% CI, −0.97 to −0.10; *p* = 0.034; *n* = 7). BMI correlated positively with cartilage volume gain (r = 0.93; 95% CI, 0.51 to 0.99; *p* = 0.006; *n* = 6), but not with WOMAC change (r = 0.49; 95% CI, −0.41 to 0.91; *p* = 0.26; *n* = 7). Platelet dose correlated negatively with WOMAC improvement (r = −0.76; 95% CI, −0.96 to −0.02; *p* = 0.047; *n* = 7) but showed no association with cartilage volume change (r = 0.04; 95% CI, −0.80 to 0.83; *p* = 0.93; *n* = 6). Finally, the estimated MSC dose did not correlate significantly with either WOMAC improvement (r = −0.57; 95% CI, −0.92 to 0.33; *p* = 0.19; n = 7) or cartilage volume change (r = 0.35; 95% CI, −0.64 to 0.91; *p* = 0.49; *n* = 6).

### 3.3. Safety, Adverse Events, and Twelve-Month Clinical/MRI Follow-Up

No technical complications occurred during US-guided bone marrow aspiration or IA BMC injection. Similarly, no clinical complications or adverse events were reported during follow-up. Adverse events were defined as:

- Pain > 3/10 within the first 7 days after the procedure.
- Symptomatic hematoma at the puncture site within 2 weeks.
- Knee or periarticular infection within 3 months.
- Tibial endplate fracture during aspiration.

None of these events were observed. One patient declined MRI follow-up at 12 months but completed clinical evaluation.

## 4. Discussion

This preliminary case series suggests that intra-articular injection of bone marrow concentrate (BMC), obtained through tibial endplate aspiration under ultrasound guidance, may provide functional benefit in patients with symptomatic patellofemoral osteoarthritis (PFOA). In most cases, WOMAC scores improved, and MRI volumetry indicated stability or even increases in cartilage volume, including one patient with a marked gain. While encouraging, these observations should be interpreted cautiously given the small sample size and exploratory nature of the study.

Our imaging results did not demonstrate statistically significant structural gains, in contrast with some prior reports of cartilage improvement following MSC-based therapies. In this study, T2 mapping was selected as a reproducible, quantitative method to assess cartilage quality while minimizing observer bias [21,22]. Only a few clinical studies to date have correlated patient-reported outcomes with imaging biomarkers of cartilage quality. Orozco et al. reported a reduction in poor-quality cartilage volume from 19.5% to 14.3% at 12 months post-injection [23], while Vega et al. observed a 69% reduction after treatment with expanded allogeneic MSCs [24]. In our series, healthy cartilage volume showed a non-significant increase, yet preservation of baseline values together with a trend toward structural stabilization could still be clinically meaningful. Considering that annual cartilage loss in OA is estimated at ~4% [25], maintenance or modest gain over 12 months may indicate slowing of degenerative progression.

When contrasted with our prior iliac crest series (Silvestre et al., JVIR 2023 [6]) (Silvestre), the present tibial endplate cohort shows clear methodological and quantitative differences while yielding comparable symptomatic signals. Demographically, the iliac crest group included 96 patients (63 men/33 women; age 53 ± 13 y; BMI 25.5 ± 3.3), whereas the tibial cohort comprised 7 patients (5 men/2 women; age 49.3 ± 9.3 y; BMI 23.1 ± 3.4). Baseline symptoms were lower in the tibial series (WOMAC 21.7 ± 17.3 vs. 37.2 ± 18.5). Product characteristics also diverged: mean injected BMC volume was 8.1 ± 2.3 mL (tibial) versus 17.7 ± 2.8 mL (iliac crest). Platelet dose in the tibial concentrate averaged 653 × 10^6^, compared with 9220 × 10^6^ ± 5813 × 10^6^ in the iliac cohort. Total leukocytes in the tibial series averaged 63.7 × 10^6^, versus 609 × 10^6^ ± 389 × 10^6^ in the iliac series. Despite these quantitative differences, both cohorts showed improvement in WOMAC scores of similar magnitude (−11 vs. −14 points). Regarding structure, iliac crest BMC significantly preserved cartilage versus untreated controls, while the tibial series showed a mean relative change of +25.4% ± 43.5% (five of six patients with stability or gain; one patient with a modest decline), which did not reach significance (*p* = 0.49). These descriptive comparisons indicate that ultrasound-guided tibial endplate aspiration yields a concentrate with distinct cellular characteristics, yet clinical and imaging outcomes were broadly aligned with those reported using iliac crest harvests.

The exploratory correlations observed in our study should be interpreted with caution due to the small sample size (n = 6–7), which limits statistical validity and increases the risk of spurious findings. While isolated associations were detected—such as a negative correlation between age and clinical improvement, or a positive correlation between BMI and cartilage gain—these results were inconsistent across outcomes, with structural and symptomatic responses not always aligned. Importantly, no correlation was found between MSC dose or CFU-F frequency and either WOMAC or cartilage volume change, which is consistent with other reports describing similar clinical improvements in patients with both high and low MSC counts, suggesting that efficacy is not linearly dependent on progenitor cell numbers [26]. Instead, therapeutic benefit likely derives from a multifactorial mechanism involving paracrine signaling, cytokines, and platelet-derived factors. This interpretation is reinforced by Park et al. (2025) [27], who emphasized that the regenerative potential of BMAC in osteoarthritis arises from a combination of mechanisms—including cartilage repair, immunomodulation, and effects on subchondral bone—rather than MSC dose alone. Collectively, these observations support the hypothesis that the clinical effect of BMC depends on the overall bioactive milieu rather than the absolute number of MSCs delivered, which may also explain why functional outcomes can be achieved even when tibial endplate aspirations yield fewer nucleated cells compared to iliac crest samples.

Based on the magnitude of clinical improvement in WOMAC scores, we calculated a Cohen’s d effect size of 0.73, which corresponds to a large effect. Following sample size recommendations summarized by Kunselman (2024) [28], a randomized controlled trial (RCT) designed to detect such an effect would require approximately 34 participants per arm for 80% power, or 44 per arm for 90% power. These estimates provide a pragmatic framework for future trial design and reinforce the need for larger, controlled studies with standardized protocols.

This study has several limitations. First, the small sample size limited statistical power, increasing the risk of type II errors and preventing detection of significant changes in cartilage volume or reliable correlation patterns. Second, the absence of a placebo or control group reduces the ability to attribute clinical improvements solely to BMC. Third, the study design was not blinded, which may have introduced expectation bias in patient-reported outcomes. Methodological limitations should also be acknowledged. Although the tibial endplate has been characterized as a feasible source of bone marrow, the specific application of ultrasound-guided aspiration with this template has not yet been validated against iliac crest harvests in terms of reproducibility, yield, and clinical outcomes. The use of ultrasound guidance enhances precision and safety, but its operator dependence and learning curve may limit generalizability. Moreover, MRI-based volumetry and T2 mapping, while objective and non-invasive, are not universally standardized, and their sensitivity to detect subtle changes in cartilage quality may be limited. Finally, variability in BMC composition across patients remains an inherent feature of autologous preparations. Although such variability can be reduced through optimized aspiration techniques and standardized processing, future studies should also include systematic cell characterization (immunophenotyping) and potency assays beyond MSC counts. Taken together, these limitations highlight the exploratory nature of this study and underscore the need for larger, controlled trials to validate both the tibial template methodology and the biological efficacy of the resulting BMC in patellofemoral osteoarthritis.

## 5. Conclusions

This preliminary study suggests that ultrasound-guided intra-articular injection of bone marrow concentrate (BMC) obtained from the tibial endplate may offer clinically relevant improvement in patients with patellofemoral osteoarthritis. The technique proved feasible, safe, and well tolerated, and it allows for simultaneous aspiration and injection without radiation exposure. While no significant gains in healthy cartilage volume were observed, the preservation of cartilage structure and the functional outcomes support the therapeutic potential of this minimally invasive approach. The observed large effect size further reinforces the clinical value of the intervention and provides a reference for the statistical design of future trials. However, variability in patient characteristics and in the cellular composition of the concentrate may complicate the interpretation of associations between product features and outcomes. Future randomized controlled trials should specifically test whether tibial endplate-derived BMC provides equivalent or superior outcomes compared with iliac crest-derived BMC, and whether it can achieve structural cartilage preservation in addition to symptomatic relief. Larger, controlled studies with standardized protocols are therefore needed to confirm these preliminary findings and to better clarify the biological mechanisms underlying the clinical response.

## Figures and Tables

**Table 1:** MR Imaging parameters.

Sequences	Parameters
Sagittal T1-weighted	TR = 588 ms, TE minimum, Nex = 1, FOV 16 cm, thickness 3 mm, spacing 0.5 mm, 24 slices, anteroposterior direction, duration 2 min 17 s
Sagittal PDW	TR/TE, 2.362/45 ms; Nex = 2, FOV 16 cm, 3 mm thickness, 0.5 spacing, 24 slices, duration 3 min 14 s
Coronal PDW	TR/TE, 2.000/45 ms; Nex = 2, FOV 16 cm, 3 mm thickness, 0.5 spacing, 20 slices, duration 2 min 44
Axial PDW	TR/TE, 2.257/45 ms; Nex = 2, FOV 16 cm, 3 mm thickness, 0.5 spacing, 24 slices, duration 3 min 05 s
Axial T2 mapping sequence for patellofemoral joint	TR = 1000 ms, TE = 6.1, 14.1, 22.1, 30.1, 38.1,46.1, 54.1, and 62.1 ms; Nex = 2; FOV 16 cm; 256 × 192 matrix; 9 slices; thickness = 3 mm with 0.6-mm spacing; duration 5 min 09 s

Note: MR imaging protocol comprised sequences included in standard protocol and T2 mapping. FOV = field of view; Nex = number of excitations; PDW = proton density-weighted; TE = echo time; TR = repetition time.

**Table 2:** Patient characteristics, bone marrow aspiration (BMA) volume, bone marrow concentrate (BMC) volume, and cell counts per patient. M: male; F: Female; MNCs: Mononucleated Cells; CFU-F: colony-forming units.

Sex	Age (Years)	Weight (kg)	Size (Meters)	BMI	BMA Volume (mL)	BMC Volume (mL)	Total Cells Injected (×10^6^)						
							MNCs	Platelets	Leukocytes	Granulocytes	Lymphocytes	Erythrocytes	CFU-F
M	51	51	1.70	17.0	20	6.0	3.36	1284	71.7	44.88	20.34	44.76	215
M	53	76	1.76	24.5	20	6.0	0.84	408	19.38	12.6	5.46	2.22	19
M	37	85	1.76	27.4	20	10	3.00	360	47.0	31.0	11.6	1.70	94
F	68	68	1.60	26.6	20	12	5.04	2040	231.5	186	38.9	25.0	1158
M	40	88	1.88	24.9	20	10	1.10	210	33.5	27.0	5.00	0.90	235
F	52	69	1.79	21.5	20	9.0	1.10	144	22.5	13.3	7.70	0.70	23
F	50	56	1.62	21.3	20	6.0	0.70	126	20.0	16.0	3.70	0.50	40
Mean ± SD	50.1 ± 10.1	70.4 ± 13.8	1.73 ± 0.10	23.3 ± 3.6	20.0 ± 0.0	8.4 ± 2.4	2.16 ± 1.66	653.1 ± 730.4	63.7 ± 76.4	47.3 ± 62.3	13.2 ± 12.7	10.8 ± 17.4	254.9 ± 408.1
Median [IQR]	51 [47.5–55.0]	69 [60.5–79.0]	1.76 [1.65–1.76]	24.5 [21.1–25.4]	20 [20–20]	9 [6–10]	1.10 [0.95–3.16]	360 [144–813]	33.5 [21.3–59.4]	27.0 [13.8–37.1]	7.7 [5.0–15.7]	1.7 [0.8–13.6]	94 [23–235]

## Data Availability

The data presented in this study are available on reasonable request from the corresponding author. The data are not publicly available due to privacy and ethical restrictions.
